# Molecular Biomarkers Linking Psoriasis and Cardiovascular Disease: Current Evidence and Clinical Perspectives

**DOI:** 10.3390/ijms27198918

**Published:** 2026-10-08

**Authors:** Mateusz Matwiejuk, Hanna Myśliwiec, Agnieszka Mikłosz, Oksana Jarocewicz, Adrian Chabowski, Iwona Flisiak

**Affiliations:** 1 Department of Dermatology and Venereology, Medical University of Bialystok, 15-540 Bialystok, Poland; 2 Department of Physiology, Medical University of Bialystok, 15-222 Bialystok, Poland; 3 Regional Centre for Transfusion Medicine, 15-950 Bialystok, Poland

**Keywords:** psoriasis, cardiovascular disease, molecular biomarkers, endocan, chemerin, growth differentiation factor-15 (GDF-15), inflammation

## Abstract

Psoriasis is a chronic, immune-mediated inflammatory disease affecting the skin, nails, and joints. Its complex pathogenesis results from interactions between genetic susceptibility and environmental factors and is accompanied by an increased risk of multiple systemic comorbidities, particularly cardiovascular disease. Growing evidence indicates that both conditions share common molecular pathways driven by chronic inflammation, endothelial dysfunction, metabolic disturbances, and immune dysregulation. Among the emerging molecular biomarkers, chemerin, endocan, and growth differentiation factor-15 (GDF-15) have attracted considerable attention due to their role in vascular homeostasis, adipose tissue biology, glucose metabolism, and inflammatory signaling. This review summarizes current evidence on the involvement of these biomarkers in psoriasis and cardiovascular disease, highlighting their contribution to the molecular mechanisms underlying the psoriasis–cardiovascular disease axis. Furthermore, we discussed their potential usefulness as candidate biomarkers for cardiovascular risk. However, prospective validation is strictly required to confirm their clinical utility and establish their incremental predictive value beyond conventional risk factors.

## 1. Introduction

Psoriasis is a complex, multifactorial condition with an immunological, genetic, inflammatory, and environmental etiology [1]. The global prevalence of psoriasis is estimated to be approximately 4.4% [2]. The pathogenesis of psoriasis is complex and involves the interplay between keratinocytes, immune cells, and other skin-resident cells. The IL-23/Th17 axis drives psoriasis by releasing key signaling proteins that cause immune cell activation and keratinocyte proliferation [3]. Clinically, psoriasis comprises several subtypes including plaque psoriasis, guttate psoriasis, palmoplantar psoriasis, scalp psoriasis, intertriginous psoriasis, nail psoriasis, erythrodermic psoriasis, and pustular psoriasis [4]. Plaque psoriasis, the most common clinical form, is characterized by well-demarcated, erythematous plaques covered with silvery scales [5]. Patients with psoriasis have an increased risk of developing numerous comorbidities, including cardiovascular disease (CVD) [6], inflammatory bowel diseases such as Crohn’s disease and ulcerative colitis [7], chronic kidney disease [8,9], end-stage renal disease [10], and metabolic disorders [11], including obesity [12], dyslipidemia [13], diabetes mellitus [14], increased waist circumference [15], and non-alcoholic fatty liver disease [16]. In addition, psoriasis is associated with an increased prevalence of psychiatric disorders, including major depressive disorder and bipolar disorder [17]. The association between psoriasis and its comorbidities is thought to be mediated, at least in part, by upregulation of the IL-23/Th17 axis [18,19,20]. Activation of this pathway promotes chronic systemic inflammation through IL-23-stimulated Th17 lymphocytes and subsequent IL-17 production, leading to immune dysregulation in multiple organs [21]. Cardiovascular diseases remain the leading cause of mortality worldwide [22]. According to the 2019 ACC/AHA Guideline on the Primary Prevention of Cardiovascular Disease, psoriasis is recognized as a cardiovascular risk-enhancing factor [23]. Psoriasis is associated with an increased risk of atrial fibrillation (AF), heart failure, MI, large artery stroke (LAS), valvular heart disease (VHD) [24], and hypertension (HTN) [25]. While epidemiologic links between psoriasis and cardiovascular disease are well established, the exact underlying molecular pathways connecting them are still being uncovered. Numerous molecular biomarkers have been proposed as potential mediators associating psoriasis and cardiovascular disease, including glycoprotein acetylation (GlycA) [26], cardiac troponins (cTnI) [27], N-terminal pro-B-type natriuretic peptide (NT-proBNP) [28], growth differentiation factor-15 (GDF-15) [29], vascular cell adhesion molecule-1 (VCAM-1) [30], S100A7A [31], S100A8/A9 [32], homocysteine [33], galectin-3 [34], zyxin [35], chemerin [36], endocan [37], and chitinase-3-like protein 1 (YKL-40) [38]. Among them, chemerin, endocan, and GDF-15 have emerged as candidate biomarkers for early detection and prognosis of cardiovascular diseases and psoriasis. Briefly, chemerin is an adipokine and chemoattractant involved in immune-metabolic regulation and recruiting immune cells (for instance, plasmacytoid dendritic cells) to the skin [39].

Endocan is an endothelial cell-derived molecule that is associated with endothelial dysfunction and systemic vascular inflammation [40].

Meanwhile, GDF-15 is a stress-responsive cytokine linked to tissue stress, metabolic control, and immunomodulatory pathways in keratinocytes [41].

Although chemerin, endocan, and GDF-15 reflect distinct biological domains—specifically inflammation, endothelial dysfunction, and tissue stress—it is hypothesized that their combined assessment could capture various aspects of the so-called “psoriatic march”. However, further research is required to determine whether their co-evaluation offers superior diagnostic or prognostic accuracy compared to the individual biomarkers. Moreover, it should be emphasized that these molecules are merely potential biomarkers, and their actual clinical utility depends on future validation in prospective studies and the demonstration of their added prognostic value over conventional cardiovascular risk factors.

The review summarizes current evidence on the role of chemerin, endocan, and growth differentiation factor-15 in the pathogenesis of both cutaneous psoriasis and cardiovascular diseases.

**Figure 1:**
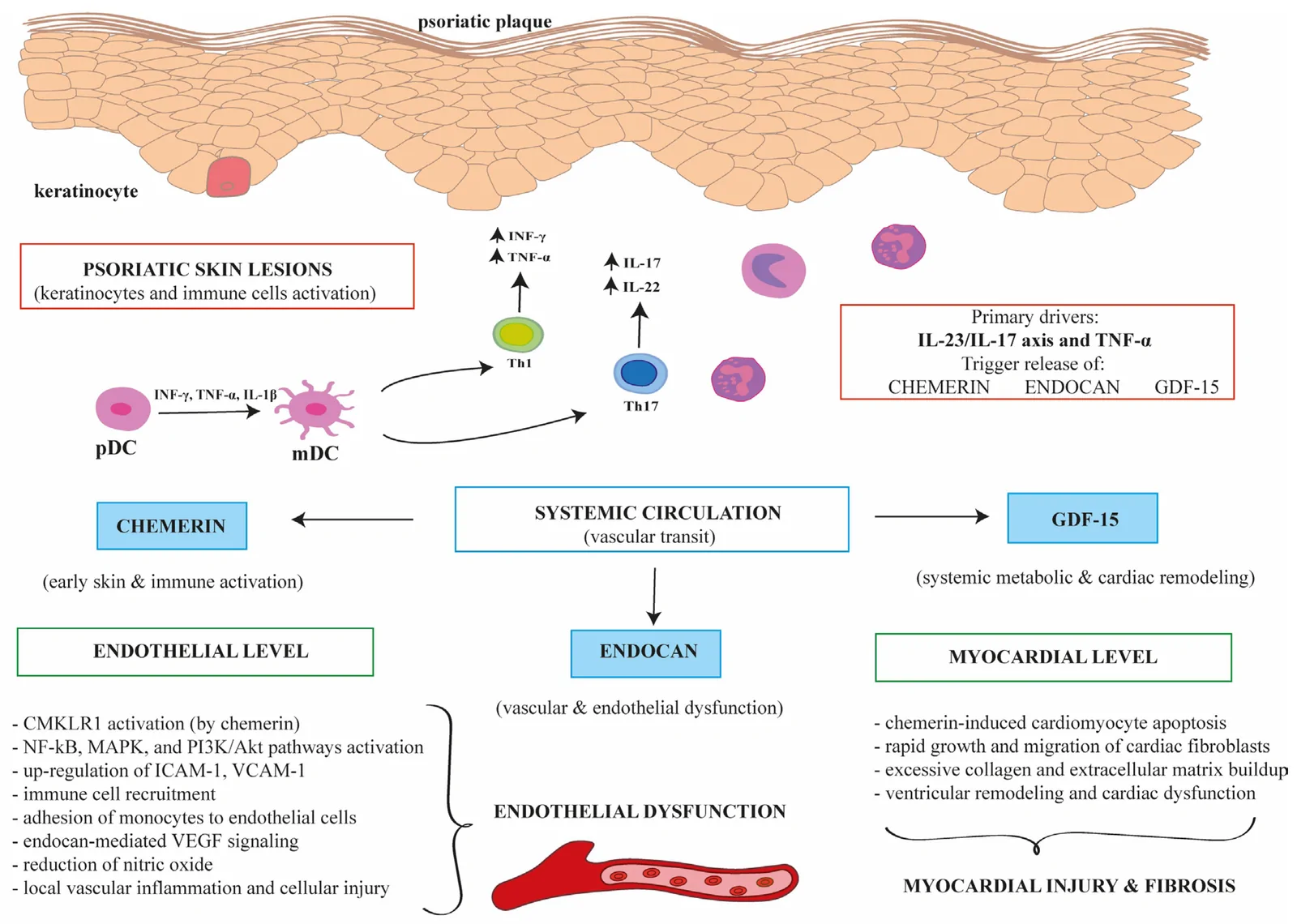
Multi-organ figure illustrating the cytokine cascade (conceptual model, which has not been clinically validated). IFN-γ—interferon-gamma; IFN-α—interferon-alpha; IL-17—interleukin-17; IL-22—interleukin-22; Th1—T-helper 1; Th17—T-helper 17; IL-23—interleukin-23; pDC—plasmacytoid dendritic cells; mDC—myeloid dendritic cells; IL-1b—interleukin-1 beta; CMKLR1—chemokine-like receptor 1; NF-kB—nuclear factor kappa-light-chain-enhancer of activated B cells; MAPK—mitogen-activated protein kinase; PI3K/Akt—phosphoinositide 3-kinase/protein kinase B; ICAM-1—intercellular adhesion molecule-1; VCAM-1—vascular cell adhesion molecule-1; VEGF—vascular endothelial growth factor; GDF-15—growth differentiation factor-15.

Figure 1 illustrates the cytokine cascade originating from psoriatic skin lesions, its dissemination through the systemic circulation, and the downstream signaling pathways mediated by chemerin, endocan, and GDF-15 at the endothelial and myocardial levels.

## 2. Discussion

### 2.1. Chemerin

Chemerin is a pro-inflammatory adipokine involved in adipogenesis, angiogenesis, inflammation, and glucose metabolism [42]. Growing evidence suggests that chemerin plays an important role in psoriasis pathogenesis by modulating keratinocyte function and inflammatory responses. Chemerin promotes keratinocyte proliferation and enhances the production of pro-inflammatory cytokines implicated in psoriasis pathogenesis. In chemerin-stimulated HaCaT cells, IL-1β, IL-6, TNF-α, IL-22, LCN2, S100A9, S100A8, and S100A7 expression levels increased significantly. In addition, chemerin activates the MAPK signaling pathway, which plays a crucial role in psoriasis pathogenesis. Chemerin binding to chemokine-like receptor 1 initiates intracellular signaling cascades in human HaCaT keratinocytes. Furthermore, it activates GTPases and kinases, which subsequently enhance mitogen-activated protein kinase (MAPK) pathways, including extracellular signal-regulated kinase 1/2 (ERK1/2), p38 MAPK, and c-Jun N-terminal kinase (JNK). ERK1/2 and p38 modulate activator protein 1 (AP-1) and downstream mediators such as MSK1/2, which subsequently phosphorylate CREB and facilitate NF-κB transactivation. MAPK-mediated recruitment of AP-1 and NF-kB complexes to the promoters of TNF and IL-1β stimulates robust mRNA synthesis. Additionally, ERK and p38 activation similarly upregulates IL6 transcription. Once translated and secreted, these downstream gene products (IL-1β, IL-6, and TNFα) can bind back to their receptors on HaCaT cells [43].

Moreover, treatment with a neutralizing anti-chemerin antibody reduced dermal inflammatory infiltration and psoriatic epidermal hyperplasia, while also decreasing the mRNA expression levels of IL-6, IL-22, IL-1β, TNF-α, and LCN2 [44]. These findings indicate that chemerin contributes to both epidermal hyperproliferation and chronic cutaneous inflammation, two hallmarks of psoriasis.

Beyond the skin, chemerin exerts multiple effects on the cardiovascular system. It influences the vascular wall through several mechanisms, including activation within the vascular lumen [45], modulation of endothelial cell adhesion [46], regulation of adipogenesis [47], and involvement in glucose metabolism and glucose homeostasis [48]. In cardiomyocytes, TNF-α increases chemerin expression and promotes apoptosis by activating protein kinase B (AKT) and caspase 9 [49]. Moreover, in cardiac fibroblasts, chemerin stimulates AKT, ERK1/2, and reactive oxygen species (ROS) production [50]. Furthermore, in endothelial cells, chemerin promotes angiogenesis and enhances ROS generation [51,52,53]. Collectively, these findings suggest that chemerin contributes to cardiovascular disease through endothelial dysfunction, vascular inflammation, atherosclerosis, and metabolic dysregulation. Elevated circulating chemerin levels have been associated with increased body mass index (BMI), impaired glucose metabolism, elevated low-density lipoprotein (LDL) cholesterol, and hypertriglyceridaemia, all of which are well-established cardiovascular risk factors [54]. In patients with psoriasis, chemerin may therefore represent an important molecular link between chronic systemic inflammation and cardiovascular disease by promoting immune activation, metabolic dysfunction, endothelial injury, and atherosclerotic processes [42]. Taken together, the pleiotropic biological functions of chemerin suggest that it may serve not only as a potential biomarker but also as a molecular factor bridging chronic cutaneous inflammation with cardiovascular dysfunction in patients with psoriasis.

Chemerin is a molecule that still requires prospective validation and demonstration of incremental value beyond conventional risk factors.

### 2.2. Endocan

Endocan is a circulating endothelial cell-specific proteoglycan primarily expressed by the vascular endothelium. It could serve as a promising potential marker of angiogenesis, inflammation, and malignancy [54]. Several pro-inflammatory cytokines and growth factors upregulate its expression, including TNF-α, IL-1β, fibroblast growth factor-2 (FGF-2), and vascular endothelial growth factor (VEGF) [55,56,57,58,59,60], whereas interferon-γ (IFN-γ) suppresses endocan expression [61].

In patients with psoriasis, circulating endocan levels are significantly elevated compared with healthy controls. Moreover, serum endocan concentrations are positively correlated with Psoriasis Area and Severity Index (PASI) scores (r = 0.477, *p* = 0.009), high-sensitivity C-reactive protein (hsCRP) levels (r = 0.484, *p* = 0.008) [37], and carotid intima-media thickness (cIMT) (r = 0.408, *p* = 0.02) [37,62]. These findings suggest that endocan reflects both systemic inflammation and subclinical vascular injury in patients with psoriasis.

#### 2.2.1. Endocan in Hypertension

Endocan has been implicated in the development and progression of HTN, primarily through its close association with endothelial dysfunction [63,64]. Elevated circulating endocan levels have been reported in patients with both HTN and coronary artery disease (CAD) compared with patients with HTN alone (1.63 ± 0.51 ng/mL vs. 1.31 ± 0.65 ng/mL, *p* < 0.05) [65]. The proposed mechanism underlying this association involves hypertension-induced endothelial injury, which stimulates endocan production while reducing nitric oxide (NO) bioavailability. Impaired NO production contributes to endothelial dysfunction and diminished vasodilation, thereby promoting the development and progression of CAD [65,66]. Several studies have also demonstrated significantly higher serum endocan levels in patients with HTN than in healthy controls [67]. However, the relationship between endocan concentrations and HTN severity remains controversial. In contrast to previous reports, Celik et al. [68] found no significant differences in serum endocan levels between patients with stage 1 and stage 2 essential hypertension, despite significantly higher endocan concentrations in hypertensive patients compared with healthy individuals (34.2 ± 13.0 vs. 24.1 ± 7.3 ng/mL, respectively; *p* < 0.001). Moreover, elevated serum endocan levels were associated with increased aortic wall thickness, further supporting its relationship with vascular injury [68].

Agac et al. [69] did not observe significant differences in serum endocan concentrations between asymptomatic hypertensive patients with or without asymptomatic target organ damage (AOD) [69], suggesting that endocan may not reliably reflect early hypertensive organ involvement. The conflicting findings between Celik et al. and Agac et al. regarding target organ damage may be due to key methodological and clinical differences, including differences in patient age distribution, disease duration, and antihypertensive washout periods. This could explain how differences in study design and baseline clinical characteristics account for the divergent conclusions between the two cohorts.

In contrast, Oktar et al. [70] demonstrated a significantly high positive correlation between serum endocan levels and both systolic and diastolic blood pressures. Furthermore, systolic blood pressure was identified as an independent predictor of endocan concentrations (*p* < 0.001), with significant positive correlations between serum endocan levels and both systolic and diastolic blood pressure [70].

Taken together, the available evidence indicates that endocan is closely associated with endothelial dysfunction and vascular injury in hypertension. Although its relationship with disease severity remains inconsistent across studies, elevated endocan levels appear to reflect vascular inflammation and endothelial activation, supporting its potential role as a biomarker associating psoriasis with increased cardiovascular risk.

#### 2.2.2. Endocan in Heart Failure

Beyond hypertension, endocan has also been implicated in the pathogenesis and prognosis of heart failure (HF), primarily through its association with endothelial dysfunction. Kosir et al. [71] reported significantly higher serum endocan levels in patients with HF than in those with CAD or healthy controls. Elevated endocan concentrations were associated with reduced left ventricular ejection fraction (LVEF), increased N-terminal pro-B-type natriuretic peptide (NT-proBNP), and New York Heart Association (NYHA) functional class (hazard ratio (HR), 1.471; 95% CI, 1.183–1.829; *p* = 0.001). Furthermore, increased endocan levels were associated with a poorer prognosis in chronic HF. Specifically, each 1 ng/mL increase in serum endocan concentration was associated with an approximately 1.5-fold higher risk of adverse HF outcomes [71]. These findings suggest that endocan may serve as a valuable biomarker for risk stratification and disease severity assessment in patients with HF.

Similarly, serum endocan concentrations were significantly elevated in patients with acute HF (AHF). Endocan levels showed positive correlations with NT-proBNP (r = 0.51, *p* < 0.001), lactic acid (r = 0.55, *p* = 0.02), and degree of left ventricular systolic dysfunction, indicating its association with HF severity [72].

In patients with end-stage HF, plasma endocan levels were significantly higher than in healthy controls (*p* < 0.001). Following heart transplantation (HT), endocan levels decreased significantly (*p* < 0.01). Moreover, this reduction was strongly correlated with reductions in pulmonary arterial pressure, pulmonary arterial wedge pressure, and pulmonary artery vascular resistance (*r*_s_ = 0.80, *p* < 0.0001; *r*_s_ = 0.63, *p* = 0.0012; *r*_s_ = 0.70, *p* < 0.001, equivocally) [73]. These observations suggest that endocan may not only reflect disease severity but also serve as a potential biomarker for monitoring treatment response, particularly in patients with pulmonary hypertension undergoing advanced HF therapy.

#### 2.2.3. Endocan in Coronary Artery Disease

Growing evidence suggests that endocan may serve as a useful biomarker for monitoring the development and progression of coronary artery disease CAD [71]. Several studies have demonstrated significantly higher serum endocan concentrations in patients with CAD than in healthy controls [74,75,76]. Elevated circulating endocan levels are thought to reflect endothelial injury and persistent vascular inflammation accompanying the progression of atherosclerosis [77]. Similarly, patients with chronic stable angina exhibit significantly higher endocan levels than healthy individuals [78,79]. Moreover, endocan has been identified as an independent predictor of well-developed coronary collateral circulation. The authors describe a strong positive correlation of serum endocan and vascular cell adhesion molecule-1 (VCAM-1) (r = 0.73, *p* < 0.001), whereas an inverse correlation was found between endocan and hs-CRP serum concentration (r = −0.35, *p* = 0.001) [78]. Endocan links psoriasis and coronary artery disease via endothelial dysfunction, which is a biomarker [37]. These findings further support the potential role of endocan as a marker of endothelial activation and vascular remodeling in CAD.

#### 2.2.4. Endocan in Atherosclerosis

Several studies have demonstrated significant positive correlations between serum endocan concentrations and cIMT, a well-established marker of subclinical atherosclerosis (r = 0.496, *p* < 0.001) [80] (r = 0.469, *p* < 0.001) [81].

Lv et al. [80] additionally reported significant positive correlations between endocan levels, the duration of type 2 diabetes mellitus (T2DM), and fasting blood glucose concentrations, whereas negative correlations were observed with high-density lipoprotein (HDL) and total cholesterol [80].

Similarly, Icli et al. [81] demonstrated positive correlations between serum endocan concentrations and BMI (r = 0.373, *p* = 0.013) and erythrocyte sedimentation rate (ESR) (r = 0.393, *p* = 0.008). The authors suggested that endocan level, as a marker of endothelial dysfunction, may help predict the progression of atherosclerosis in patients with systemic lupus erythematosus (SLE) [81].

Elevated serum endocan levels have also been associated with subclinical atherosclerosis in chronic obstructive pulmonary disease (COPD) [82] and Behçet disease [83]. Pihtili et al. [82] observed significantly higher endocan concentrations in patients with COPD than in the healthy controls (860.1 ± 259.9 vs. 647.3 ± 316.9 pg/mL, *p* = 0.001) [82]. In these studies, subclinical atherosclerosis was assessed using cIMT measurements, which provide a non-invasive marker of early arterial wall thickening before the onset of clinically overt cardiovascular disease [84].

#### 2.2.5. Endocan and Antihypertensive Drugs

Treatment with amlodipine has been shown to improve endothelial dysfunction, as reflected by reduced serum endocan and CRP levels in patients with HTN. However, changes in serum endocan concentrations were not directly correlated with the magnitude of blood pressure reduction [85].

Endothelial activation in HTN is regulated, at least in part, by interactions between soluble cell adhesion molecules and circulating leukocytes, in which endocan is thought to play an important regulatory role. Amlodipine was associated with significant reductions in serum soluble vascular cell adhesion molecule-1, soluble intercellular adhesion molecule-1, and endocan concentrations, indicating attenuation of endothelial activation. These changes were accompanied by lower blood pressure values, suggesting that the vascular benefits of amlodipine may extend beyond its antihypertensive effects to include improvement of endothelial function [86].

To sum up, it could be concluded that observed reductions in endocan are primarily attributed to the mechanical deactivation of the endothelium resulting from lower blood pressure, rather than a direct anti-inflammatory drug action.

#### 2.2.6. Endocan and Hypolipidemic Drugs

Treatment with rosuvastatin (40 mg/day) following acute MI was associated with a significant reduction in serum endocan concentrations, from 110.73 (77.28–165.22) pg/mL to 93.40 (70.48–115.13) pg/mL (*p* = 0.014) [87]. The decrease in circulating endocan levels was accompanied by attenuation of endothelial dysfunction, vascular inflammation, and neointimal formation, suggesting that endocan may reflect the beneficial vascular effects of statin therapy during the progression of atherosclerosis [88].

Similarly, simvastatin reduced endocan expression in lipopolysaccharide (LPS)-stimulated endothelial cells. In addition, simvastatin reversed LPS-induced suppression of NO production and reduced ROS generation, further supporting its protective effects on endothelial function [89].

Collectively, these findings suggest that endocan is not only a promising biomarker of endothelial dysfunction and vascular inflammation but also a dynamic marker responsive to pharmacological interventions. Changes in circulating endocan levels following antihypertensive and lipid-lowering therapies highlight its potential utility for monitoring vascular response and evaluating the efficacy of cardiovascular treatment.

Regarding lipid levels, the observed reductions in endocan are generally considered to reflect direct pleiotropic, anti-inflammatory, and endothelial-stabilizing statin actions rather than being solely secondary to drops in lipid levels. However, it is a molecule which still requires prospective validation and demonstration of incremental value beyond conventional risk factors.

### 2.3. Growth Differentiation Factor-15 (Gdf-15)

Growth differentiation factor-15 (GDF-15) belongs to the family of transforming growth factor β (TGF-β) [87,90,91]. GDF-15 possesses multiple physiological and pathological functions [92,93,94]. GDF-15 plays an important role in cancer cell growth and proliferation [95]. Interestingly, at early stages in healthy tissue, it stops carcinogenesis and promotes tumor progression at final stages. GDF-15 acts via p53- or EGR-1-related pathways. Moreover, elevated levels of NAG-1 halt the AKT/GSK-3beta pathway [96]. GDF-15 also possesses anti-inflammatory features. Deficiency of GDF-15 leads to dysregulation of the interleukin-6-dependent inflammatory response to vascular damage and impaired regulation of apoptosis [97]. In healthy pregnant women, GDF-15 enhances insulin secretion, preventing the development of pregnancy-induced insulin resistance [98]. In addition, it was concluded that GDF-15 could serve as a potential biomarker of miscarriage, fetal growth restriction [99,100,101], preeclampsia [102], and gestational diabetes [103,104,105]. In cardiac muscle, GDF-15 is primarily recognized for its cardioprotective properties, in detail, by reducing cardiomyocyte death, dilation, and hypertrophy of cardiac muscle [106,107]. In addition, it was shown that GDF-15 expression was boosted by ROS, ischemia, volume overload, and reperfusion injury [108]. GDF-15 stimulates angiogenesis in the vascular wall, abnormal endothelial cells, and proliferation of muscle cells [109,110]. Interestingly, GDF-15 prevents atherosclerotic plaque disruption and limits macrophage accumulation [111,112]. Upregulation of GDF-15 leads to a decrease in cardiac inflammation and apoptosis [113]. Activation of PI3K/AKT and SMAD2/3 signaling causes a reduction in cardiomyocyte apoptosis, decreasing inflammatory cell infiltration and curbing the risk of cardiac rupture [114]. In HF and cardiac hypertrophy, the enhancement of the SMAD2/3 pathway attenuated cardiomyocyte hypertrophy [106]. Moreover, it was observed that GDF-15 was correlated with decreased levels of plasma growth hormone (GH), suggesting that GDF-15 might be involved in the GH signaling pathway. Higher circulating GDF-15 levels were noted in patients with failure to thrive, congenital heart disease, and cardiomyopathy [115]. GDF-15 was revealed as a stronger predictor of cardiac mortality than NT-proBNP (HR 1.5 [1.3 to 1.8], *p* < 0.0001 for GDF-15 vs. 1.3 [1.2 to 1.5], *p* < 0.0001 for NT-proBNP) [116]. In another study, GDF-15 was significantly correlated with three cardiac conditions: atherosclerotic CVD (HR 1.43; 95%CI: 1.20–1.58), HF (HR 2.08; 95%CI: 1.72–2.53), and cardiac mortality (HR 1.96; 95%CI: 1.76–2.17) [117].

In ACS, patients with elevated GDF-15 levels were prone to bleeding risk (HR 1.37, 95% CI 1.25–1.51), stroke (HR 1.19, 95% CI 1.01–1.42), death due to CVD (HR 1.41, CI 1.30–1.53), and MI (HR 1.15, 95% CI 1.05–1.26) [118]. In the study conducted by Tzikas et al. [119], the authors observed a significantly higher GDF-15 level in patients who underwent MI compared to patients with non-MI (967.1 pg/mL vs. 692.2 pg/mL, *p* < 0.001). Higher GDF-15 concentrations at hospital admission were associated with an increased risk of MI [119]. Bodde et al. [120] found that GDF-15 was associated with 10-year all-cause mortality (HR 2.453 (95% CI 1.064–5.658), *p* = 0.04) in ST-segment elevation myocardial infarction (STEMI) patients treated with primary percutaneous coronary intervention (pPCI). In addition, GDF-15 was positively and significantly correlated with NT-proBNP (0.17, *p* = 0.004) and not significantly correlated with cardiac troponin T (cTnT) (0.083, *p* = 0.191) [120]. Rueda et al. [121] described progression within 24 h in STEMI, and GDF-15 concentrations peaked 12 h after the cardiac event (1731 pg/mL, *p* < 0.001) and remained higher at 24 h. GDF-15 was a strong predictor of 30-day mortality (HR 1.76, CI 1.33–2.34) [121]. GDF-15 was an independent marker of left ventricular remodeling (LVR) in patients who suffered STEMI (OR 10.1, 95% CI 2.5–40.1, *p* < 0.001) and treatment with angiotensin-converting enzyme inhibitors (ACEIs) (OR 3.9, 95% CI 1.2 to 12.3, *p* < 0.01) [122]. Lindholm et al. [123] observed a three-fold risk of bleeding in patients who underwent ACS (one month after) and took ticagrelor and clopidogrel, who had an elevated level of GDF-15 (>1800 ng/L), regardless of drug intake [123]. GDF-15 is elevated in both systolic and diastolic heart failure, is an independent risk predictor of adverse effects and mortality, and is independent of ejection fraction and serum level of NT-proBNP [124,125]. In patients with heart failure with preserved ejection fraction (HFpEF) versus reduced ejection fraction (HFrEF), GDF-15 was similarly elevated (HFpEF [2862 (1812, representing the 25th percentile and 4176 representing the 75th percentile) ng/L] and HFrEF [2517 (1555–4030) ng/L] (*p* = 0.184)) and served as a prognostic factor. In comparison, NT-proBNP was significantly decreased in HFpEF in contrast to HFrEF (1119 ng/L vs. 2335 ng/L, *p* < 0.001) [126]. In another study, it was revealed that patients suffering from HFPEF and HFREF were characterized by higher median concentrations of GDF15 (540 pg/mL vs. 2529 and 2672 pg/mL, respectively). GDF15 distinguished HFPEF from controls with an area under the curve (AUC) of 0.936 (*p* < 0.001). Moreover, it was concluded that the ratio of NT-proBNP:GDF15 acts as a distinguishing factor between HFPEF and HFREF, with the strongest AUC (0.709; *p* < 0.001) [127]. Patients dealing with a severe stage of HF were older than the other group and had the highest tertile of GDF-15. Additionally, GDF-15 was also described as a risk factor for all-cause death in patients with HF (HR 2.03, *p* < 0.0001) [128]. Elevated levels of GDF-15 were correlated with a higher risk of developing left atrial thrombus in comparison to patients without left atrial thrombus and CHA_2_DS_2_VASc [129]. GDF-15 has been proposed as one of the most valuable potential biomarkers incorporated into the ABC (age, biomarkers and clinical history) bleeding risk score [130].

#### Gdf-15 in Psoriasis

GDF-15 may correlate with the severity of the PASI score. Moreover, it was observed that serum GDF-15 levels > 1498.5 pg/mL may point to a raised PASI score. Patients suffering from psoriasis were divided into three separate groups regarding PASI scores: group 1—PASI < 10 (mild stage of psoriasis); group 2—PASI 10–20 (moderate stage of psoriasis); and group 3—PASI > 20 (severe stage of psoriasis). The authors noticed a significantly positive correlation between PASI score (in all three groups) and GDF-15 value in the psoriatic patients (*p* < 0.001) [131].

GDF-15 levels in psoriatic serum and gene expression were significantly elevated in the study group compared with the control group and were positively correlated with the severity of psoriasis. The average GDF-15 serum level in mild, moderate, and severe cases of psoriasis was 0.45 ± 0.35, 2.27 ± 0.7, and 3.5 ± 1.6 ng/mL. Moreover, the mean of GDF-15 gene expression in the mild, moderate, and severe forms of psoriasis was 5.25 ± 3.2, 7.6 ± 2.8, and 17.8 ± 5.7. Based on their study, the researchers concluded that the GDF-15 protein could serve as a potential biomarker of psoriasis [132].

Serum GDF-15 levels were significantly raised along with TNF-α in psoriatic patients in comparison to healthy controls (*p* < 0.001 and *p* = 0.015, respectively). In addition, a positive correlation was noticed between GDF-15 and TNF-α levels (r = 0.455, *p* = 0.04). No correlation was found between GDF-15 and PASI score [133]. Interestingly, the GDF-15 value was induced by IL-6, TNF-α, hypoxia, and oxidative stress [134,135].

In another study, patients who were suffering from psoriasis had higher levels of GDF-15 in their serum compared to the control group, and it was positively correlated with psoriasis duration and PASI score (*p* < 0.001, respectively) [29]. Interestingly, in contrast to the above-mentioned finding, one proposed mechanism for GDF-15 in the pathogenesis of psoriasis is its anti-inflammatory action, whereby it acts as a beta-2 integrin antagonist. Consequently, it traps leukocytes on the endothelium and prevents their infiltration into the inflammatory site [136].

It was revealed that imiquimod-treated GDF-15 knockout (GDF-15 -/-) mice had more severe psoriatic skin lesions than wild-type mice. Conversely, treatment with recombinant GDF-15 improved psoriasis symptoms, indicating that GDF-15 possesses anti-inflammatory properties [137].

Importantly, Jiang et al. [138] performed a Mendelian randomization analysis using the fixed-effects inverse-variance weighted (IVW) method and demonstrated an inverse association between genetically anticipated GDF-15 levels and psoriasis risk (OR = 0.88, 95% CI: 0.82–0.95, *p* = 2.58 × 10^−4^) [138].

While GDF-15 upregulation is strongly associated with active disease states like psoriasis and cardiovascular injury, experimental evidence often paints its local upregulation as a compensatory, protective (anti-inflammatory and anti-apoptotic) tissue response to stress rather than a primary driver of injury. Current clinical data cannot prove a direct causal involvement of GDF-15 in the psoriasis–cardiovascular axis. Instead, elevated levels likely represent a systemic epiphenomenon—a shared molecular “distress signal” reflecting background chronic inflammation, metabolic dysfunction, and vascular aging.

GDF-15 is a molecule that still requires prospective validation and demonstration of incremental value beyond conventional risk factors. 

In summary, an overview of candidate biomarkers for cardiovascular risk evaluation in psoriatic patients is provided in Table 1.

### 2.4. Biologics Impact on Chemerin, Endocan, and Gdf-15

Fassio et al. [143] showed in their clinical trial that secukinumab (anti-IL-17A) possessed a predominantly neutral effect on total systemic adipokines, like chemerin, over 6 months. In this prospective, uncontrolled study, the researchers evaluated a small group of 28 patients with psoriatic arthritis, classified based on the CASPAR criteria [143].

Currently, there is a lack of randomized controlled trials evaluating the effects of biologic therapies for psoriasis on endocan and GDF-15 levels.

## 3. Limitations and Future Perspectives

Chemerin, endocan, and GDF-15 are general markers of systemic inflammation, endothelial dysfunction, and cellular stress. Because their levels rise in numerous inflammatory conditions, malignancies, and metabolic disorders, it is highly challenging to isolate the specific contributions of psoriasis versus subclinical cardiovascular disease. The majority of studies evaluating endocan, GDF-15, and chemerin in psoriatic patients are cross-sectional or observational. These designs can only establish statistical associations between biomarkers and surrogate parameters (such as carotid intima-media thickness or plaque burden), failing to prove a causal link or define true prognostic value.

Currently, there are no universally validated reference intervals or standardized cutoff values for circulating GDF-15 and endocan in dermatological and cardiometabolic practice. Variations in enzyme-linked immunosorbent assay (ELISA) kits and analytical platforms hinder the comparability of values across independent cohorts. Furthermore, psoriasis frequently coexists with components of the metabolic syndrome—such as obesity, insulin resistance, and hypertension—which independently disrupt the regulation of circulating chemerin and GDF-15. Consequently, it is difficult to precisely determine the independent impact of psoriatic skin inflammation on cardiovascular risk. Moreover, GDF-15 and endocan levels are influenced by renal clearance dynamics; undiagnosed or subclinical renal impairment can significantly alter their circulating concentrations, falsely elevating systemic levels independently of primary cutaneous disease severity or vascular damage.

Large-scale, long-term prospective studies should monitor patients with psoriasis over several years to evaluate whether tracking changes in chemerin, endocan, or GDF-15 can accurately predict critical cardiovascular endpoints (e.g., myocardial infarction, stroke) prior to clinical manifestation. Additionally, targeted clinical trials should investigate how biological therapies (such as IL-17, IL-23, or TNF inhibitors) affect the trajectories of these biomarkers. This will clarify whether chemerin, endocan, and GDF-15 can serve as reliable surrogate tools for therapeutic monitoring to track the simultaneous resolution of skin and vascular inflammation.

## 4. Materials and Methods

A comprehensive literature search was performed in PubMed, Google Scholar, and Embase covering the period from 1996 to 2026. The primary objective was to identify and evaluate the role of the selected proteins—chemerin, endocan, and growth differentiation factor-15 (GDF-15)—in the pathogenesis of psoriasis and their association with cardiovascular disease. To provide broader context, the search strategy also included studies addressing psoriasis, cardiovascular diseases, hypolipemic treatment, and hypertensive treatment.

The following Medical Subject Headings (MeSH) terms and keyword combinations were used: “chemerin”, endocan”, “GDF-15”, “psoriasis”, cardiovascular diseases”, “biomarker”, and “inflammation”. Original studies involving humans and animal models related to molecular, immunological, and clinical aspects of psoriasis and cardiovascular diseases were included. Publications written in languages other than English, studies of low clinical relevance, and duplicates were excluded. After de-duplication, titles and abstracts were filtered by three reviewers (M.M., A.M., H.M.). Disagreements were resolved through consensus between the same reviewers. Full texts of eligible articles were then assessed in detail.

## 5. Conclusions

Growing evidence indicates that endocan, chemerin, and GDF-15 are potential molecular biomarkers bridging psoriasis with cardiovascular disease. Beyond reflecting systemic inflammation, these proteins participate in key pathogenic mechanisms shared by both conditions, including endothelial dysfunction, vascular remodeling, immune dysregulation, adipogenesis, glucose metabolism, and atherosclerosis. Although their biological functions differ—GDF-15 predominantly exerts anti-inflammatory effects, endocan acts mainly as a marker of endothelial activation and inflammation, and chemerin displays both pro- and anti-inflammatory properties—all three molecules provide valuable insight into the complex interplay between chronic skin inflammation and cardiovascular risk.

While these biomarkers show promise for future cardiovascular risk stratification models, they currently lack immediate clinical utility. Research evaluating endocan, GDF-15, and chemerin in psoriasis patients is limited by predominantly cross-sectional study designs and a lack of standardized cutoff values or reference intervals. Confounding factors such as coexisting metabolic syndrome and unrecognized renal impairment further complicate the precise isolation of cardiovascular risk stemming from psoriatic skin inflammation. Therefore, their routine clinical application requires further validation in large prospective studies, together with the standardization of reliable assays and the establishment of clinically relevant reference ranges. A better understanding of the molecular mechanisms involving these biomarkers may also facilitate the development of novel targeted therapeutic strategies for patients with psoriasis and its cardiovascular comorbidities.

## Figures and Tables

**Table 1:** Overview of candidate biomarkers for cardiovascular risk evaluation in psoriatic patients. All listed molecules are currently considered candidate biomarkers; most evidence is derived from cross-sectional cohorts or non-psoriatic populations and lacks proven incremental prognostic value over traditional risk scores.

Biomarker	Biological Function (Mechanistic)	Evidence in Psoriasis	Evidence in Cardiovascular/Mechanistic Diseases	Clinical Relevance	Current Limitations
Chemerin	Involvement in adipogenesis, angiogenesis, inflammation, glucose metabolism [42]; innate and adaptive immunity (recruiting plasmacytoid dendritic cells and neutrophils into psoriatic plaques) [39].	Involvement in creating plaque psoriasis and correlation with PASI score [139]. Acts as a pivotal chemoattractant and pro-inflammatory adipokine that drives the initiation of plaque psoriasis through its correlation with the PASI score. Chemerin directly enhances the proliferation of epidermal keratinocytes.	It promotes monocyte-endothelial cell adhesion, drives early atherosclerotic plaque formation, and correlates heavily with endothelial dysfunction, arterial stiffness, and coronary artery disease severity [42]. Activation within the vascular lumen [45]; modulation of endothelial cell adhesion [46]; regulation of adipogenesis [47].	Contributes to cardiovascular disease through endothelial dysfunction, vascular inflammation, atherosclerosis, and metabolic dysregulation [42]. Assessment of psoriasis severity [39].	Not enough large prospective studies; it still requires prospective validation and demonstration of incremental value beyond conventional risk factors.
Endocan	Involvement in angiogenesis, inflammatory processes, and malignancy [54]. It reduces nitric oxide bioavailability which leads to endothelial dysfunction and diminished vasodilation [65,66]; its expression correlates with acanthosis and parakeratosis [139]; endocan regulates cell adhesion pathways (such as the LFA-1/ICAM-1 pathway) [140].	Correlated with PASI score [37]. It is a marker to flag early, subclinical vascular damage (which requires validation) such as increased CIMT in psoriasis patients who do not yet show overt cardiovascular disease [37,62].	Promoting CAD [71]; positive correlations with NT-proBNP, lactic acid, and degree of left ventricular systolic dysfunction [72]; elevated circulating endocan levels reflect endothelial injury; persistent vascular inflammation accompanying the progression of atherosclerosis [77].	Reflects both systemic inflammation and subclinical vascular injury in patients with psoriasis [37]. Marker of endothelial dysfunction, plaque instability, and major adverse cardiac events (MACEs) [141].	Large prospective studies are still lacking; endocan requires prospective validation and demonstration of incremental value beyond conventional risk factors.
GDF-15	Cancer cell growth, cancer proliferation [95]; angiogenesis; proliferation of abnormal endothelial cells and of muscle cells [109,110]; abnormal epidermal GDF15 boosts keratinocyte cytokine and chemokine synthesis by not blocking the TAK1/NF-κB signaling pathway [142].	Correlation with PASI score [29,130].	Reduction in cardiomyocyte apoptosis, decreased inflammatory cell infiltration, curbing the risk of cardiac rupture [114]; it is strongly upregulated by pro-inflammatory cytokines (like TNF-α, IL-6), hypoxia, and oxidative stress [134,135].	GDF-15 may correlate with the severity of the PASI score [29,131,132]; GDF-15 was significantly correlated with atherosclerotic CVD, HF, and cardiac mortality [117]. It is a prognostic indicator for heart failure, myocardial infarction, and mortality (especially in patients with cardiovascular diseases) [120,121,124,125].	Not enough large prospective studies; it still requires prospective validation and demonstration of incremental value beyond conventional risk factors.

## Data Availability

No new data were created or analyzed in this study. Data sharing is not applicable to this article.

## Abbreviations

The following abbreviations are used in this manuscript:

AHF	Acute heart failure
AOD	Asymptomatic target organ damage
AF	Atrial fibrillation
AUC	Area under the curve
cTnI	Cardiac troponins
CVDs	Cardiovascular diseases
cIMT	Carotid intima-media thickness
YKL-40	Chitinase-3-like protein 1
COPD	Chronic obstructive pulmonary disease
CAD	Coronary artery disease
FGF-2	Fibroblast growth factor-2
GlycA	Glycoprotein acetylation
GDF-15	Growth differentiation factor-15
HR	Hazard ratio
HT	Heart transplantation
HF	Heart failure
HFrEF	Heart failure with reduced ejection fraction
HFpEF	Heart failure with preserved ejection fraction
hsCRP	High-sensitivity C-reactive protein
HTN	Hypertension
IVW	Inverse-variance weighted
LAS	Large artery stroke
LVEF	Left ventricular ejection fraction
LVR	Left ventricular remodeling
MACEs	Major adverse cardiac events
MI	Myocardial infarction
NYHA	New York Heart Association
NT-proBNP	N-terminal pro-B-type natriuretic peptide
NO	Nitric oxide
OR	Odds ratio
pPCI	Primary percutaneous coronary intervention
ROS	Reactive oxygen species
STEMI	ST-segment elevation myocardial infarction
SLE	Systemic lupus erythematosus
TGF-β	Transforming growth factor β
VHD	Valvular heart disease
VCAM-1	Vascular cell adhesion molecule-1
VEGF	Vascular endothelial growth factor
